# Absorption of Medicines by the Skin

**Published:** 1867-12

**Authors:** 


### EDITORIAL DEPARTMENT.

*Absorption of Medicines by the Skin*—We published recently some results of experiments on absorption in which the various surfaces were considered in reference to their capacity of admitting medicinal agents into the circulation. In the paper of which we gave an abstract, it was assumed that iodine made its way into the circulation through the mucous membrane of the bronchial tree, because so little was taken up from solution.

A series of experiments has recently been performed by Dr. Roussin, still further illustrative of cutaneous absorption. After immersion of an hour or an hour and a half in a bath containing 500 grammes (1 pound $1\frac{5}{8}$ ounces of av.,) of iodide of potassium he detected no trace of iodine in the urine. If, however, he suffered the solution to evaporate spontaneously upon his skin iodine soon made its appearance in the urine. It was never taken up from solution but always in the dry state, when thin from the evaporation of the solution or from friction with the dry powder.

Dr. Roussin finds the reason for this in the physical condition of the cutaneous surfaces. This is always more or less oily and no amount of soaping effects more than a temporary abstersion of this natural grease for the simple reason that the sebaceous glands are constantly forming fatty secretion. Hence water will not easily wet the skin, but extends over it in drops. Of course, this greasiness repels the water, and with it the substances it holds in solution, the pores of the skin following the universal rule of all capillary tubes. It is now easy to see why mercury and iodine are so readily introduced into the circulation when applied as unguents. The fat which contains them finds pores in the exact condition required for its admission. So too the dry powder rubbed on the surface forms a sort of unguents with the free fat, and then readily passes into the face.

